# Skin cancer risk affected by ultraviolet solar irradiance in Arica, Chile

**DOI:** 10.3892/ol.2013.1698

**Published:** 2013-11-22

**Authors:** MIGUEL RIVAS, ELISA ROJAS, GLORIA M. CALAF

**Affiliations:** 1 Department of Physics, Faculty of Sciences, Tarapaca University, Arica 8097877, Chile; 2 Institute for Advanced Research, Tarapaca University, Arica 8097877, Chile; 3 Center for Radiological Research, Columbia University Medical Center, New York, NY 10032, USA

**Keywords:** skin, cancer, risk, ultraviolet light

## Abstract

The present study analyzed the risk of skin cancer due to ultraviolet erythemal irradiance (UV_ery_) in Arica, Chile, using measurements of the solar ultraviolet index (UVI) between 2006 and 2011. The daily maximum value by biometer Yankee Environmental Systems (YES) solar ultraviolet B (UVB)-1 was measured between 2007 and 2012, and seasonal variations were clearly observed, with higher UVI levels during the summer when UVI usually reached extreme values of >11. The maximum UVI value was 15.6 in the summer of 2008 and the minimum was 2.2 in the winter of 2008. The UVI mean values that were collected monthly at noon between 2006 and 2010 fluctuated between 13 and 6, and reached higher values in January and lower values in June and July. Thus, a seasonal UVI response was observed during the two seasons. The accumulated UV_ery_/day was calculated between September 2006 and 2007, the time when Arica normally receives the highest UVI levels. It was also noted that 60% of the days in September demonstrated values of >3.41 kJ/m^2^/day, while 3.3% of cloudy days had values of <2.0 kJ/m^2^/day. The mean value of UV_ery_ during 2007 was 3.23 kJ/m^2^/day and the variation was 1.9–4.6 kJ/m^2^/day. These UV_ery_ values were several times higher than the minimal erythemal doses (MEDs) corresponding to the skin types most frequently observed in Chile, skin types III and IV. The MED for skin type IV was 0.60 kJ/m^2^. The results demonstrated that the skin cancer rate was increased due to the fact that individuals from Arica are exposed to several times more UV_ery_ than the MED for their skin type during the spring and summer seasons.

## Introduction

The incidence of skin cancer per 100,000 inhabitants significantly increased in Arica from 17.6 to 31.4 between 2000 and 2007 (1). This rate is high compared with other cities in Chile.

Skin exposure to solar ultraviolet B (UVB) in the range of 290–320 nm has multiple consequences that may be dangerous for human beings (2). Overexposure is known to induce skin cancer and immune suppression. The most frequently affected skin type in Chile is type III in females and type IV in males (3), according to the Fitzpatrick skin type classification (4). The minimal erythemal dose (MED) is the minimum amount of UV that produces redness 24 h after exposure. Skin type III corresponds to skin that burns and tans moderately and uniformly (MED, 0.30–0.50 kJ/m^2^), and skin type IV corresponds to light brown skin that burns minimally and tans moderately and easily (MED, 0.40–0.60 kJ/m^2^). Lighter skin transmits more radiation and consequently, the dose and the time that is necessary to induce erythema is less than for darker skin. The present study aimed to assess the risk of exposure of individuals to the sun according to the skin types, as individuals in Arica, Chile, receive ultraviolet erythemal irradiance (UV_ery_) that is several times greater than the MED for the two skin types.

## Materials and methods

UVI measurements were collected for one minute every day between 2006 and 2011 using Biometer Yankee Environmental Systems (YES) UVB-3 calibrated accordance with World Meteorological Organization guidance. The UV_ery_ levels were calculated between September 2006 and December 2007. The data were considered to be representative of the typical spring UVI radiation in this location. The instrument was mounted on a building with an unobstructed view and involved a set of radiometric instruments, including radiometers and photometers. UVI measurements were performed using a YES-UVB-1 ultraviolet biometer (YES Inc., Turners Falls, MA, USA) that was regularly calibrated and used in agreement with the Direction of Meteorology of Chile (DMC). This instrument integrates the ultraviolet network of Chile and is calibrated according to the recommendations of the WMO.

The time taken in minutes to produce erythema (t_ery_) in unprotected skin is calculated from the following equation:

$$t_{ery}=\frac{MED(skin_{type})}{UV_{ery}}$$

UV_ery_ was directly obtained through the equation:

$$UV_{ery}=\frac{UVI}{40}\left(\frac{mW}{m^{2}}\right)$$

## Results

**Figure 1:**
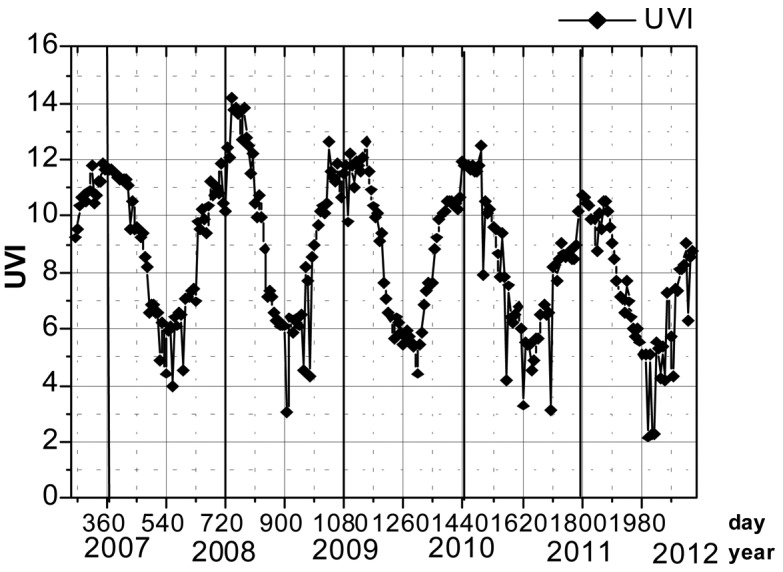
Solar ultraviolet index (UVI) mean values measured by biometer Yankee Environmental Systems solar ultraviolet B (UVB)-1 instrument between 2007 and 2011 in Arica, Chile.

The present study provides a general overview of the UVI that has been reported in the city of Arica, Chile, and obtained using a YES biometer of a UV laboratory in agreement with the DMC from a country with a latitude of 18°49′S. The daily maximum UVI measured by biometer YES UVB-1 in Arica between 2007 and 2012 is shown in Fig. 1. Seasonal variations were clearly observed, with high UVI levels during the summer, which reached extreme values of >11 according to the WMO classification. The results also demonstrated that the maximum UVI that was measured in this period was recorded as 15.6 in the summer of 2008. The minimum value was 2.2 in the winter of 2008.

**Figure 2:**
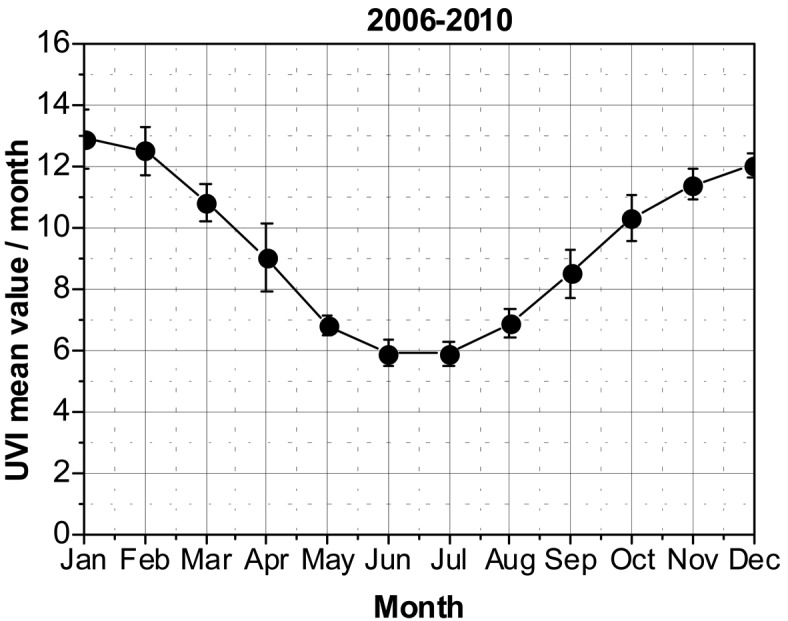
Solar ultraviolet index (UVI) mean values per month measured by biometer Yankee Environmental Systems solar ultraviolet B (UVB)-1 instrument between 2006 and 2010 in Arica, Chile.

**Figure 3:**
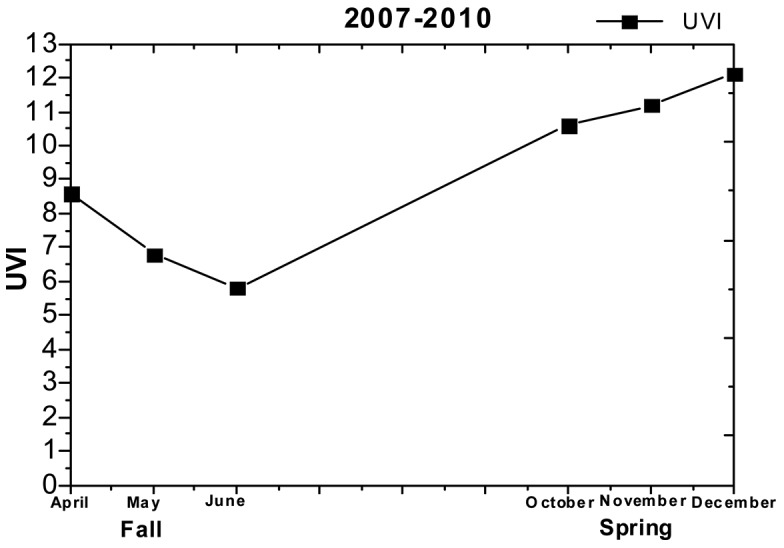
Solar ultraviolet index (UVI) mean values/month measured by biometer Yankee Environmental Systems solar ultraviolet B (UVB)-1 between April and June (fall season), and October and December (spring season), between 2007 and 2010 in Arica, Chile.

The UVI mean values/month were collected daily at noon in Arica between 2006 and 2010. The UVI values fluctuated between 13 and 6 and reached the highest values in January and the lowest in June and July, as shown in Fig. 2. The UVI mean values/month were analyzed between 2007 and 2010, between April and June in the fall season and between October and December in the spring season in Chile (Fig. 3).

**Figure 4:**
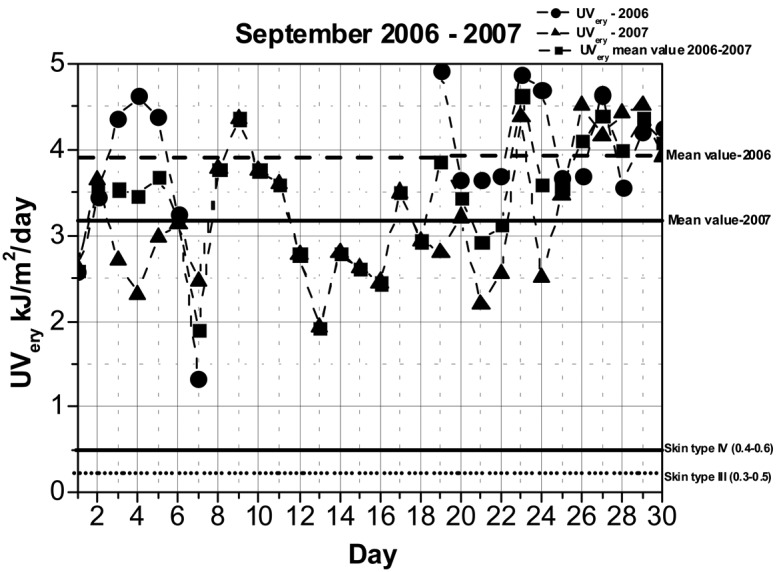
Mean and daily ultraviolet erythemal irradiance (UV_ery_) values to ultraviolet irradiance kJ/m^2^/day between September 2006 and 2007. Bold and dotted lines indicate the minimum erythemal doses (MEDs) to skin types IV and III.

**Figure 5:**
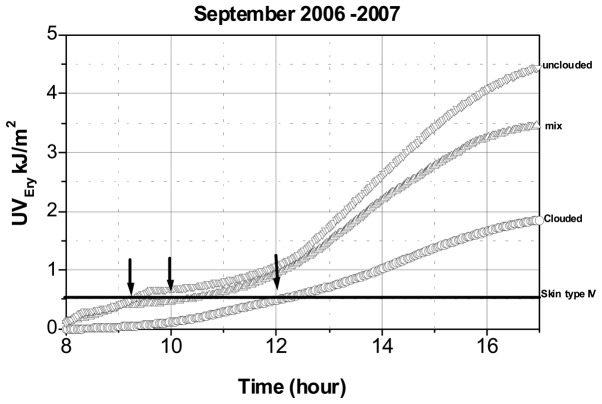
Daily ultraviolet erythemal irradiance (UV_ery_) every 5 min during the clear, clouded and a mixture of the two types of day between September 2006 and 2007. Bold line indicates minimum erythemal doses (MEDs) to skin type IV (0.40–0.60 kJ/m^2^).

Fig. 4 shows the accumulated UV_ery_/day that was obtained between September 2006 and 2007. The mean value of UV_ery_ during 2007 was 3.23 kJ/m^2^/day. The results indicated that the fluctuation in UV_ery_ during this period was 1.9–4.6 kJ/m^2^/day. These values were several times higher than the MED corresponding with skin types III and IV. In Fig. 5, the daily UV_ery_ values that were obtained every 5 min during the clear, clouded and a mixture of the two types of day between September 2006 and 2007 are shown. A non-linear increase was observed over the day, and the black line indicated that the MED for skin type IV was 0.60 kJ/m^2^. It is of note that when the curve corresponded to a clear day, it reached the black line at 9:00 AM. Therefore, the UV_ery_ received by individuals during the rest of the day was higher than the MED for skin type IV. The daily accumulated UV_ery_ between 2006 and 2007 fluctuated between 1.90–4.63 kJ/m^2^/day. It is noteworthy that 60% of the days in September exhibited values of >3.41 kJ/m^2^/day, while cloudy days were responsible for 3.3% of the days, with values of <2.0 kJ/m^2^/day. The monthly mean value of erythemal UV_ery_ during September was 3.41±0.70 kJ/m^2^/day. Table I shows the mean UV_ery_ that was reached at various daily time periods between September 2006 and December 2007. The data show a high risk of receiving excessive UV_ery_ for individuals who were exposed at the beach for 2 h between 11:00 AM–13:00 PM, when individuals with skin types III and IV received UV_ery_ of between 1.7- and 3.5-fold greater than the MED. Whereas for the same period of time in the evening between 16:00–18:00 PM, when individuals with MED skin types III and IV were exposed, UV_ery_ was 0.8–1.5-fold greater than the MED. In the time period between 8:00 AM–17:00 PM, when skin type III and IV were exposed, UV_ery_ varied by 6.4–12.9-fold.

## Discussion

The present study provides a general overview of the skin cancer risk affecting individuals who live in the city of Arica, Chile, which is located in the subtropical zone of northern Chile (25 meters above sea level, 18°49′S latitude and 70°19′W longitude) and presents a micro-weather with stable spring type meteorological conditions throughout the year, including a lack of rain (>5 mm/decade), predictable types of winds, a high percentage of clear days and a high reflectivity of the ground, due to mainly light sand. The city is located at sea level, therefore, the population perform a great number of activities outside their homes. The high accumulated UV_ery_ measured in Arica is contributed by various factors, including closeness to the equator, lack of green areas, highly reflective surfaces (sand and ocean), and local ozone minimum values measured in this location during spring and summer seasons.

The present study showed the UVI measurements that were obtained using a biometer YES UVB-1. Seasonal variations are evident, with high UVI levels being observed during the summer when the UVI usually reaches values of >11, according to the classification of the WMO. The maximum UVI that was identified in this period was 15.6 during summer 2008 and the minimum was 2.2 during winter 2008.

The UVI mean values per month that were collected at noon in Arica between 2006 and 2010 indicated that the UVI ranged between 6 and 13, reaching higher values in January and lower values in June and July. The seasonal response in UVI and ozone layer thickness demonstrated an inverse correlation of these variables for the two seasons. Accumulated UV_ery_/day recorded between September 2006 and 2007 revealed that the mean value was 3.9 kJ/m^2^/day, and the erythemal dose interval during this period was 1.3–5.0 kJ/m^2^/day. The UV_ery_ reported in the present study was several folds higher than the MEDs of 0.30–0.50 and 0.40–0.60 kJ/m^2^, corresponding with skin types III and IV, respectively (5). It has previously been reported that ultraviolet light exposure affects skin cancer in association with latitude (6).

The daily UV_ery_ was determined every 5 min during one clear, one clouded and one with a mixture of the two types of day between September 2006 and 2007, and a non-linear increase was observed during the day. The red line indicated that the MED was 0.40 and 0.60 kJ/m^2^ for skin type IV. It is notable that the curve reached the red line at 10:30 AM, following which, UV_ery_ appeared to be dangerous to the individuals with skin type IV over a long-term exposure, particularly for those that had been exposed during the rest of the day.

UV_ery_ calculated in various days between September 2006 and 2007 fluctuated between 4.91–1.33 kJ/m^2^/day. It is notable that 50% of those days had values of >3.87 kJ/m^2^/day, while cloudy days represented 5.6% of the days with values <2.0 kJ/m^2^/day. The monthly mean ± standard deviation of the UV_ery_ value during September was 3.87±0.87 kJ/m^2^/day, for which the cloudy days may possibly be responsible. The results of the present study are important for predicting the effect of exposure of the health of individuals who live in the northern part of Chile. The detailed daily information that individuals receive with regard to the IUV in this city is important, as individuals are overexposed to high UV solar radiation for the majority of the year, and this information is in accordance with the constant increase in skin cancer rates/100,000 that have been recorded in this region since 2001. In conclusion, the overexposure to UV solar radiation has multiple consequences to the health of individuals in this region and may be dangerous to human beings. The results demonstrated that the skin cancer rate increased due to the fact that individuals from Arica are exposed to UV_ery_ several times greater than the MED for their skin type during the spring and summer seasons.

## Figures and Tables

**Table I:** Daily mean erythemal reactivity to UV irradiance and exposure times for MED skin type III (0.30–0.50 kJ/m^2^/day) and MED skin type IV (0.40–0.60 kJ/m^2^/day) during September 2006.

Exposure	UV_ery_, kJ/m^2^/day	MED skin type III	MED skin type IV
Sun beach exposure (11:00 AM–13:00 PM)	1.044	2.09–3.48	1.74–2.61
Lunch time exposure (12:30 PM–13:00 PM)	0.324	0.65–1.08	0.54–0.81
Sun exposure at the beach (after 16:00 PM)	0.451	0.90–1.50	0.75–1.13
Sun beach exposure (10:00 AM–16:00 PM)	3.101	6.20–10.34	5.17–7.75
Soccer game exposure (16:00 PM–18:00 PM)	0.456	0.91–1.52	0.76–1.14
All day exposure (8:00 AM–17:00 PM)	3.866	7.73–12.89	6.44–9.67

Rates of erythemal ultraviolet (UV) solar irradiance and MED were obtained by dividing UV_ery_ and values of MED skin type III 0.30–0.50 kJ/m^2^/day and skin type IV 0.40–0.60 kJ/m^2^/day. MED, minimal erythemal dose; UV_ery_, ultraviolet erythemal irradiance.
